# Tumor Microenvironment in Male Breast Carcinoma with Emphasis on Tumor Infiltrating Lymphocytes and PD-L1 Expression

**DOI:** 10.3390/ijms24010818

**Published:** 2023-01-03

**Authors:** Iva Brcic, Andrea Maria Kluba, Theresa Marie Godschachner, Christoph Suppan, Peter Regitnig, Nadia Dandachi, Sigurd Friedwald Lax, Marija Balić

**Affiliations:** 1 Diagnostic and Research Institute of Pathology, Comprehensive Cancer Centre, Medical University of Graz, 8010 Graz, Austria; 2 Division of Oncology, Department of Internal Medicine, Medical University of Graz, 8036 Graz, Austria; 3 Research Unit for Epigenetic and Genetic Cancer Biomarkers, Medical University of Graz, 8036 Graz, Austria; 4 Department of Pathology, Hospital Graz II, Academic Teaching Hospital of the Medical University Graz, 8020 Graz, Austria; 5 School of Medicine, Johannes Kepler University Linz, 4020 Linz, Austria; 6 Unit for Translational Breast Cancer Research, Medical University of Graz, 8036 Graz, Austria; 7 Subcenter for Breast Care, Comprehensive Cancer Center, Medical University of Graz, 8036 Graz, Austria

**Keywords:** male breast cancer, intrinsic subtypes, pan-TRK, TILs, PD-L1, HER2-low

## Abstract

Male breast cancer (MBC) is rare and usually presents as a locally advanced disease. Stromal tumor-infiltrating lymphocytes (sTILs) are associated with a better response to neoadjuvant chemotherapy and improved prognosis in all molecular subtypes of female breast cancer, but their role in MBC is less clear. We studied sTILs and the expression of programmed cell death ligand 1 (PD-L1) and pan-TRK in MBC. We retrospectively studied 113 cases of MBC surgically treated between 1988 and 2015. The tumors were evaluated for histological type and grade, stage, intrinsic subtype and sTILs. We performed immunohistochemistry for PD-L1 (clone SP142) and pan-TRK (clone EPR17341) on tissue microarrays. Pan-TRK positive cases were further analyzed by next-generation sequencing. The median age was 69 years (range 60–77). Invasive carcinoma of no special type was found in 94.7% of cases, of which 53.1% were grade 2. Estrogen receptor was positive in 92% of the tumors, progesterone receptor in 85.8%, androgen receptor in 70.8%; 4.4% were human epidermal growth factor receptor 2 (HER2)-positive, and 55.8% HER2-low. 40.7% of tumors were luminal A and 51.3% luminal B, 4.4% HER2-enriched and 3.5% triple negative carcinoma. sTILs density was <50% in 96.4% of the tumors, >50% in 3.6% of the tumors. PD-L1 immune cell score >1% was found in 7.1% of the tumors (all of luminal subtype). A weak focal cytoplasmic pan-TRK staining was present in 8.8% but without *NTRK* fusion. Neither sTILs nor PD-L1 had statistically significant outcomes. Our findings suggest that a subset of MBC patients harbors an immunological environment characterized by increased sTILs with PD-L1 expression. These patients may potentially benefit from immune checkpoint inhibitor therapy. Frequent HER2-low may offer novel anti-HER2 treatment options.

## 1. Introduction

Male breast cancer (MBC) is rare and accounts for around 1% of all diagnosed breast cancers [1,2]. In males, due to the proximity of the breast tissue to the skin, MBC often presents as a locally advanced disease with regional lymph node metastasis. On the molecular level, 10–16% of MBC shows mutations in BRCA2, 4.1% in CHEK2 and 1–4% in BRCA1, suggesting differences in the molecular pathogenesis of male and female breast cancer [2,3]. In contrast to female breast carcinoma (FBC), there is evidence that the distribution of the intrinsic subtypes is also different in MBC, with a strong predominance of the luminal A and B subtypes [4,5].

The advanced stage at diagnosis requires adjuvant or neoadjuvant systemic therapy in most MBCs. A majority of MBCs are estrogen receptor (ER) positive and, therefore, treated with systemic endocrine therapy. In contrast, human epidermal growth factor receptor 2 (HER2) seems infrequently overexpressed in MBC and, therefore, not targetable. Consequently, other putative therapeutic approaches are of key interest, including the immunological tumor microenvironment, immune checkpoints and oncogenic gene fusions. Recent studies show that stromal tumor-infiltrating lymphocytes (sTILs) are predictive of the tumor’s response to neoadjuvant chemotherapy in all molecular subtypes of FBC [6], but their role in MBC, including the effect on prognosis, is less clear. Interactions between tumor and immune cells via programmed cell death 1/programmed cell death receptor ligand 1 (PD-1/PD-L1) pathway and the cytotoxic T-lymphocyte antigen 4 (CTLA4) system are potential therapeutic targets in many solid cancers. A PD-L1 cross-link of tumor cells with T-lymphocytes expressing PD-1 leads to T-cell anergy, as well as the promotion and activation of regulatory T cells (Tregs) and allows tumor cells to evade the host immune system and inhibit the immune response. Immunotherapy directed against PD-1 or PD-L1 has gotten lasting responses in different solid tumors, including FBC, but has not yet been well studied in MBC, [7,8].

Another target that has recently been identified in different cancer types, including colon cancer and lung cancer, and which can be therapeutically approached by selective small-molecule inhibitors of tyrosin receptor kinases (TKI), are fusions of the neurotrophin receptor genes (NTRK) 1–3 [9,10]. Pan-TRK immunohistochemistry (IHC) is a reliable, highly sensitive, but less specific diagnostic surrogate marker used to identify *NTRK* fusion, though it needs to be confirmed by molecular techniques, particularly next-generation sequencing (NGS) [9,11,12]. The frequency of NTRK fusions in MBC is currently unknown.

We retrospectively studied a large consecutive cohort of MBC for clinicopathological and immunohistochemical features, including therapeutical aspects and follow-up. Additionally, we analyzed the tumor microenvironment, including sTILs and PD-L1 IHC, as well as its prognostic relevance. Finally, we analyzed the MBC cohort for *NTRK* fusions using pan-TRK IHC as a screening tool.

## 2. Results

### 2.1. Study Population and Histopathological Characterization

A total of 113 male patients with a median age of 69 years (25th–75th percentile: 60–77 years, range 29–92) were included in this study. The most frequent histological type was invasive breast carcinoma of no special type (NST) (107/113, 94.7%), followed by invasive lobular carcinoma (4/113, 3.5%), mucinous carcinoma (1/113, 0.9%) and tubular carcinoma (1/113, 0.9%). Histopathological grading scored 10 tumors at grade 1 (8.9%), 69 tumors at grade 2 (53.1%) and 43 tumors at grade 3 (38.0%).

**Figure 1:**
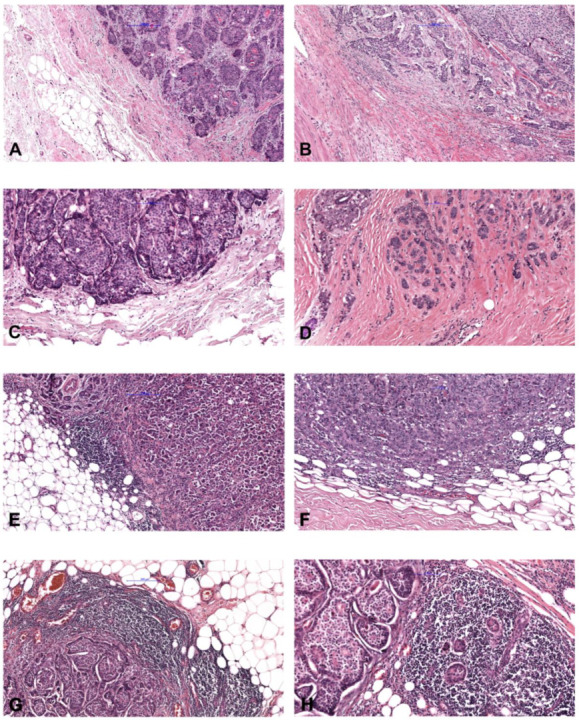
sTILs evaluation within the borders of the invasive tumor. 0–10% ((**A**–**D**); magnification (**A**,**B**) ×50, (**C**) ×300, (**D**–**F**) ×400), >10–50% (**E**,**F**) and >50% (**G**,**H**) of the stromal area shows a mononuclear infiltrate.

Immunohistochemical data and sTILs evaluation are summarized in Table 1. The majority of cases were positive for ER (92.1%), PR (85.8%), and AR (70.8%), whereas HER2 (4.4%) positivity was uncommon. However, 55.8% of the tumors were HER2-low, including 50 score 1+ tumors and 13 score 2+ tumors without amplification. The median Ki-67 labeling index was 20% (25th–75th 10–35%). Intrinsic subtypes comprised 40.7% luminal A and 51.3% luminal B carcinomas, 4.4% HER2-enriched carcinomas and 3.5% triple-negative carcinoma. Most tumors revealed a minimal to mild sTILs density of ≤50% (109/113, 96.4%, Figure 1A–C), and only four tumors (3.6%) had sTILs density >50% (Figure 1D).

**Figure 2:**
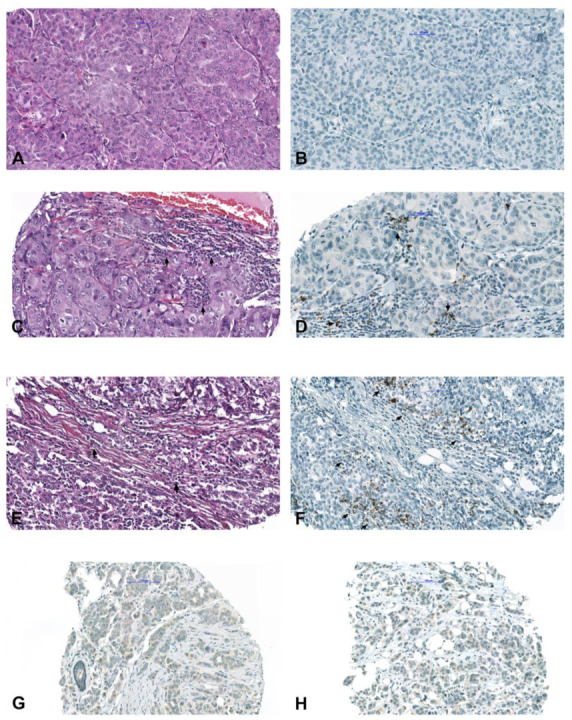
Immunohistochemical findings for PD-L1 and pan-TRK. (**A**). Invasive breast cancer without tumor-infiltrating immune cells (IC, magnification ×400) and (**B**) negative PD-L1 staining (magnification ×250. (**C**,**E**) Invasive breast cancer with focal ((**C**) magnification ×335) and scattered ((**E**) magnification ×250) tumor-infiltrating IC (arrows). (**D**,**F**) PD-L1 shows focal dark brown punctate staining in intratumoral single-IC or small aggregates (arrows) dispersed in >1% of tumor (magnification ×380). (**G**,**H**) Tumor cells show weak cytoplasmatic pan-TRK expression (magnification ×200).

**Figure 3:**
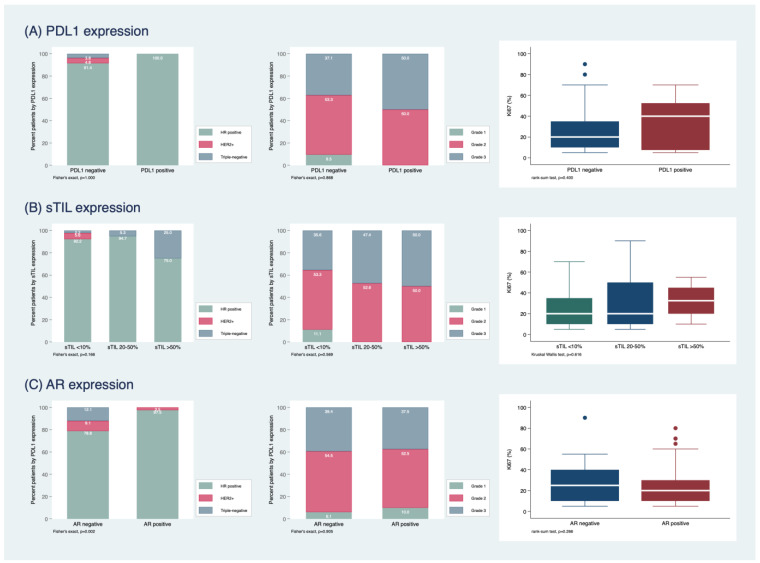
Associations between (**A**) PD-L1 (**A**,**B**) stromal TILs (sTILs) and (**C**) AR expression with breast cancer subtype, tumor grade and Ki67 index. Colored dots in the boxplot are outside values beyond the upper or lower whisker.

PD-L1 positivity (>1%) was found in 8/113 (7.1%) of the tumors (Figure 2A–F). The association between PD-L1 and sTIL, respectively, and histopathological tumor characteristics are shown in Figure 3. Pan-TRK showed weak focal cytoplasmic staining in 10/113 (8.9%) tumors (Figure 2G–H); however, NGS did not detect any *NTRK* fusion.

### 2.2. Clinical Outcome

Data on treatment and follow-up were available only for 54 (47.8%) patients who received treatment at the Division of Oncology, Department of Internal Medicine, Medical University Graz. Out of these, 36 (66.7%) patients received adjuvant radiotherapy, four (7.4%) neoadjuvant chemotherapy, 11 (20.4%) adjuvant chemotherapy, one (1.9%) neoadjuvant endocrine therapy and 48 (88.9%) adjuvant endocrine therapy. In this cohort, only one patient was Her2 positive, however, at the time of diagnosis the patient did not receive anti Her2 treatment. The majority of these patients (51; 94.4%) were treated by mastectomy, and only 3 (5.6%) patients underwent breast-conserving surgery. In addition, 47 (87%) patients underwent axillary dissection, in seven (13%) patients sentinel lymph node resection was performed. Thirty-six (66.7%) patients had regional lymph node metastasis and 11 (20.4%) patients subsequently developed distant metastasis.

The median follow-up time (with follow-up truncated at 10 years) was 8.7 years. During this observation time, 18 (33.3%) patients died and 22 (40.1%) patients had a tumor recurrence. The 3, 5 and 10-year OS estimates were 92%, 79% and 52%, respectively. The 3, 5 and 10-year RFS estimates were 81%, 69%, and 45%, respectively (Supplementary Figure S1).

**Figure 4:**
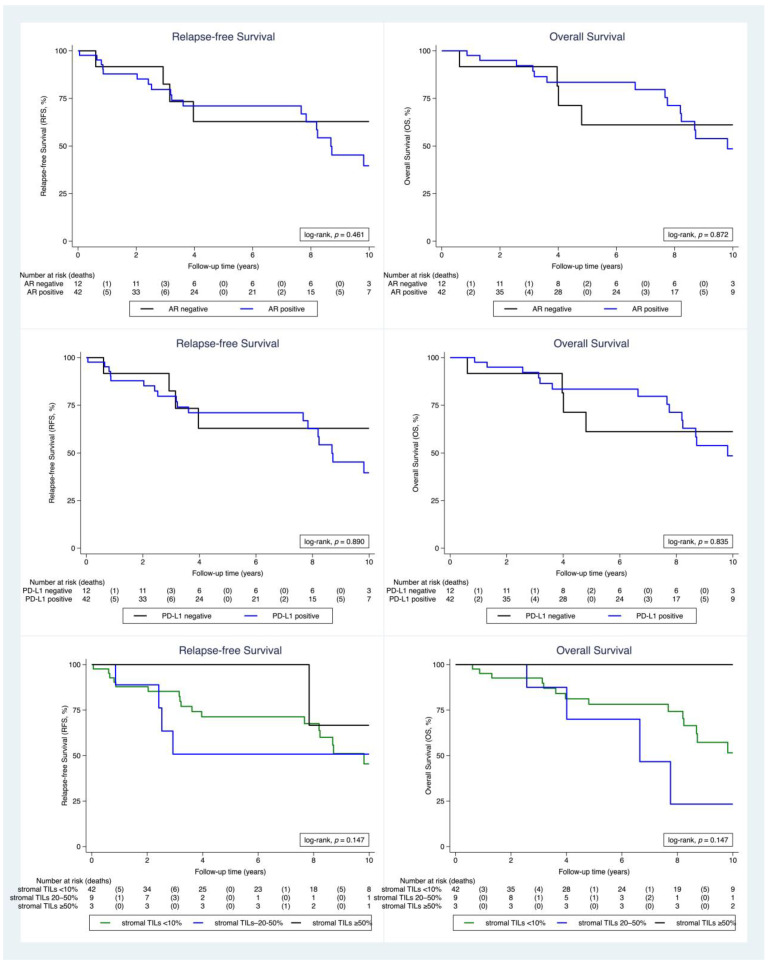
Relapse-free survival (**left panel**) and overall survival (**right panel**) according to AR, PD-L1 and stromal TILs expression in the cohort of 54 patients without distant metastasis at the time of diagnosis with available follow-up data.

The association between PD-L1, sTIL, AR, tumor type and grade and Ki-67 is summarized in Figure 4. In short, PD-L1 positive tumors were grade 2 or 3 and were all hormone receptor positive. In contrast, PD-L1 negative tumors were of all grades and included HER2-positive and TNBC as well. In addition, Ki-67 was significantly higher in PD-L1 positive tumors compared to PD-L1 negative tumors. The TNBC most commonly expressed sTILs >50%, whereas the HER2+ subgroup showed only sTILs expression <10%. Furthermore, all grade 1 carcinomas had an sTILs expression of <10. All TNBC were also AR negative. There was no significant difference between histological grade and AR expression, but Ki-67 was higher in the AR negative group. Survival analysis (Figure 4) showed a trend for better survival of tumors with sTILs >50%, but statistical significance was not reached due to the limited number of cases. Relapse-free survival for sTILs when a cut-off of 10% was used did not show any significant difference between the two groups (Supplementary Figure S2). AR and PD-L1 expression did not reveal statistical significance for survival. Finally, relapse-free survival for HER2-negative and HER2-low tumors was not statistically significant (Supplementary Figure S3).

## 3. Discussion

Our study confirms that MBC is morphologically and biologically distinct from FBC by the higher frequency of NST carcinomas and ER-positive luminal A and B carcinomas, as well as the rarity of HER2 enriched and TNBC [4,5,13,14]. We are further able to show that a subset of MBC is characterized by an immunological tumor environment characterized by increased sTILs and/or PD-L1 expression, which may be of therapeutic and prognostic relevance. In addition, we found that more than 50% of MBC in our cohort could be categorized as HER2-low, which is also of potential therapeutic value. However, we could not find an NTRK alteration by a combination of IHC and NGS.

PD-L1 is considered a biomarker for response to therapy of invasive breast carcinoma with immune checkpoint inhibitors, particularly for triple-negative FBC [15]. Tumor-infiltrating immune cells present in the intratumoral and contiguous peritumoral stroma are visualized using the SP142 assay (Ventana, Roche) and include lymphocytes, macrophages, and cells with dendritic or reticular morphology. In FBC, data about the prevalence of PD-L1 expression, as well as its relation to prognosis, are limited, and to some point controversial. In a meta-analysis, Guo et al. [16] found that strong PD-L1 expression in FBC is significantly associated with lymph node metastasis, poor nuclear grade, and negative ER status and suggested PD-L1 as a significant biomarker for poor prognosis and adverse clinicopathological features. Li et al. [17] demonstrated a high correlation between PD-L1 expression and FOXP3+Treg lymphocytes in tumor tissue, suggesting the importance of tumor evasion from the host immune system. The data on MBC are even more limited. We are unaware of a similar evaluation of MBC, neither for PD-L1 expression nor for sTILs. Thus, even though our cohort with available clinical data is small, we believe it provides important insights into the immune component of MBC. In analogy to FBC, we could even identify a small proportion of patients with high lymphocytic infiltration.

Concerning the molecular features and intrinsic subtypes [5,18,19], our results were comparable to the dataset published by Cardoso et al., which represents the most extensive available data on MBC thus far [4]. The proportion of HER2-positive MBC was slightly lower in our cohort, with 4.4% compared to 8% in the larger cohort by Cardoso et al. However, our analysis is the first reporting, particularly the proportion of HER2-low carcinomas among MBC, which is higher than in the Austrian AGMT_MBC registry [20]. These findings may be helpful for novel treatment approaches [21].

Whether MBC has other potential targets remains to be elucidated. One of the emerging targets in precision oncology has become *NTRK*, but chromosomal translocations involving the *NTRK* genes are uncommon in most solid neoplasms. These fusions have been identified in up to 0.13% of FBC, including only secretory breast cancer with *ETV6-NTRK3* fusion [9,22,23,24,25]. In non-secretory FBC, *NTRK* rearrangement was not found. Data on pan-TRK immunoreactivity and molecular analysis for *NTRK* fusions have not been published so far in MBC. In our MBC cohort, about 9% of the cases showed focal weak cytoplasmic immunoreactivity, but without a gene fusion using NGS. These findings support the current knowledge regarding the lack of *NTRK* fusion and the expression of TRK proteins in non-secretory FBC.

In conclusion, our study suggests that most MBCs are not only ER-related endocrine dependent but frequently HER2-low and characterized by an immunological, sTILs-containing tumor microenvironment. Our findings have the potential to provide further insights into the biology of the rare entity of MBC.

## 4. Materials and Methods

All consecutive adult male breast cancer patients who underwent surgery between 1988 and 2015 were retrieved from the patient files from the archives of the Diagnostic and Research (D&R) Institute of Pathology, and the Department of Pathology of the Hospital Graz II, and retrospectively studied. Formalin-fixed, paraffin-embedded (FFPE) tissue blocks and hematoxylin and eosin (H&E)-stained whole tissue sections were retrieved and reviewed by three pathologists (IB, TMG, SFL) for the histological type and grade according to 2019 WHO classification. The samples from the D&R Institute of Pathology were provided by Biobank Graz, Austria. In addition, the distribution of sTILs was evaluated, and appropriate viable tumor tissue areas for tissue microarrays (TMA) construction were selected. All cases with adequate clinical data and sufficient tumor tissue for TMA construction were included in the study. Ethical approval was obtained from the Ethics Committee of the Medical University of Graz (29–625 ex 16/17).

### 4.1. Evaluation of Stromal Tumor-Infiltrating Lymphocytes (TILs)

The assessment of sTILs was performed using the guidelines of the International TIL Working Group [26,27]. One H&E slide was evaluated per specimen. sTILs were reported as the percentage of the stromal area within the borders of the invasive tumor occupied by mononuclear immune cells (lymphocytes, plasma cells). The sTIL counts were grouped into three categories: 0–10%, 11–50% and 51–90%. Any lymphocytic infiltrate around normal lobules, at the previous biopsy site, or in areas of crush artifacts were excluded.

### 4.2. Tissue Microarray Construction

TMA construction was performed by a manual tissue microarray apparatus (Beechers Instruments, Sun Prairie, WI, USA). Three 0.6 mm cores were taken from each case and embedded in a donor paraffin block. One of the cores was selected from the invasive front of the tumor. TMAs were constructed in duplicates. For quality control, 4 μm-thick, H&E-stained sections were cut from all TMA.

### 4.3. Immunohistochemical Analysis

IHC was performed on the TMA blocks using 4 µm-thick paraffin sections. The following antibodies were used, including appropriate positive controls: ER, PR, AR, HER2, Ki-67, PD-L1 and pan-TRK (Table 2).

ER, PR and HER2 immunoreactivity were assessed using the 2018 ASCO/CAP guidelines [28]. For ER, PR and AR, the percentage of positive cells and the intensity of staining were recorded. HER2 2+ cases were analyzed additionally by fluorescence in situ hybridization (FISH). Ki-67 labeling index was assessed by counting 500–2000 tumor cells in a “hot spot” area, and the percentage of Ki-67 positive cells was calculated. For PD-L1 immunoreactivity, the immune cell score (IC) was assessed according to the interpretation guidelines for triple-negative breast cancer (TNBC). Any staining intensity in immune cells (lymphocytes, macrophages, dendritic cells and granulocytes) within the tumor or in the contiguous peritumoral stroma was considered positive when covering >1% of the tumor area. Different staining patterns included aggregates in the stroma and single cells dispersed among tumor cells with punctate, linear or circumferential staining. For pan-TRK, membranous, cytoplasmic or nuclear staining of any intensity in ≥1% of tumor cells was considered positive.

### 4.4. Molecular Analysis

All cases that were immunoreactive for pan-TRK were analyzed for specific *NTRK1*, *NTRK2*, and *NTRK3* rearrangements for the production of NTRK fusion transcripts using the Archer fusionplex sarcoma panel. Molecular analysis was performed in the Laboratory for Diagnostic Genome Analysis of the Diagnostic & Research Institute of Pathology, Medical University of Graz, Austria.

#### 4.4.1. RNA Workflow

For each case, 5 to 8 FFPE sections were cut at 10-μm from a representative FFPE tumor block and macro-dissection was performed with a scalpel to enrich the tumor content. Semi-automated RNA extraction was performed on the Maxwell RSC Instrument according to the manufacturer’s instructions, using the Maxwell RSC FFPE Plus RNA Purification Kit (AS1440, Promega, Thermo Fischer Scientific, Waltham, MA, USA).

#### 4.4.2. Targeted Next-Generation Sequencing

RNA was quantified by ribogreen fluorescence and 250 ng total of RNA was used for the Archer Fusionplex Sarcoma kit to assess specific *NTRK*1, *NTRK*2, and *NTRK*3 rearrangements. NGS libraries were sequenced on an Ion Torrent Proton using the Ion PI Hi-Q Sequencing 200 kit (Thermo Fischer Scientific). The data were analyzed with ArcherDX analysis software version 5.1.3.

### 4.5. Statistical Analysis

Statistical analysis was performed using Stata 17.0 (Stata Corp., Houston, TX, USA). Continuous variables were reported as medians (25th–75th percentile), and count data were reported as absolute frequencies (%). Median follow-up time was estimated with the reverse Kaplan-Meier estimator [29]. The association between histopathological tumor characteristics was assessed with the rank-sum test (continuous variables), chi-squared test or Fisher’s exact test (categorical variables). The association between histopathological tumor characteristics and clinical outcome was evaluated with Kaplan-Meier estimators and a log-rank test. Recurrence-free survival (RFS) was defined as a composite of local recurrence from breast cancer, distant metastasis from breast cancer or death from any cause, whichever occurred first during the follow-up period. Overall survival (OS) was defined as the time between surgery and death from any cause or censoring alive, respectively.

## 5. Conclusions

Our study suggests that most MBCs are not only ER-related endocrine dependent but frequently HER2-low and characterized by an immunological, sTILs-containing tumor microenvironment. Our findings have the potential to provide further insights into the biology of the rare entity of MBC.

## Figures and Tables

**Table 1:** Baseline patient and tumor characteristics including sTILs, PD-L1 IC score and pan-TRK expression.

		Total *n* = 113 (%)
**Age at diagnosis**		69.0 (60.0–77.0)
**Histologic grade**	Grade 1	10 (8.8%)
	Grade 2	60 (53.1%)
	Grade 3	43 (38.1%)
**ER**	Positive	104 (92%)
	Negative	9 (8%)
**PR**	Positive	97 (85.8%)
	Negative	16 (14.2%)
**HER2**	Positive	5 (4.4%)
	“low”	63 (55.8%)
	Negative	45 (39.8%)
**AR**	Positive	80 (70.8%)
	Negative	33 (29.2%)
**TNBC**		4 (3.6%)
**PD-L1**	Positive	8 (7.1%)
	Negative	105 (92.9%)
**Pan-TRK**	Positive	10 (8.8%)
	Negative	103 (91.2%)
**sTILs**	<10	90 (79.6%)
	10–50	19 (16.8%)
	>50	4 (3.6%)

Data are represented as medians (25th–75th percentile) for continuous variables, and as frequencies (%) for count data. Legend: AR—androgen receptor; ER—estrogen receptor; PR—progesteron receptor; sTILS—stromal tumor-infiltrating lymphocytes; TNBC—triple-negative breast cancer.

**Table 2:** Antibodies used for immunohistochemical analysis.

Antibody	Source	Clone	Dilution
ER	Dako/Agilent	EP1	RTU
PR	Dako/Agilent	PgR 1294	RTU
AR	Dako/Agilent	AR441	1:50
Ki-67	Dako/Agilent	MIB-1	RTU
HER2	Dako/Agilent	Hercep Test	RTU
PD-L1	Roche/Ventana Medical Systems	SP142	1:50
Pan-TRK	Roche/Ventana Medical Systems	EPR17341	RTU

Legend: AR—androgen receptor; ER—estrogen receptor; PR—progesterone receptor; RTU—ready to use.

## Data Availability

Not applicable.

## Supplementary Materials

The following supporting information can be downloaded at: https://www.mdpi.com/article/10.3390/ijms24010818/s1.
