# Supplementary material for: Tumor Microenvironment in Male Breast Carcinoma with Emphasis on Tumor Infiltrating Lymphocytes and PD-L1 Expression
Source: Int J Mol Sci. 2023 Jan 3;24(1):818. doi: 10.3390/ijms24010818 (PMC9821263; doi:10.3390/ijms24010818)
Supplement: Supplementary file 1 [file ijms-24-00818-s001.zip › ijms-2020346-supplementary.pdf]

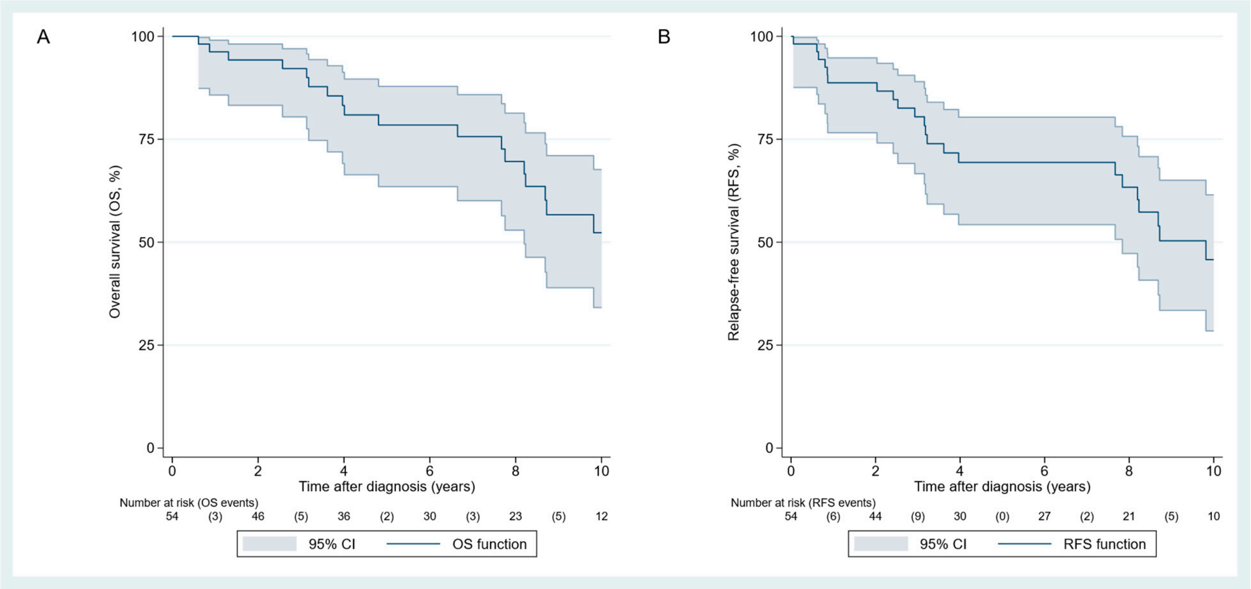

Supplementary Figure S1. Overall survival (A) and recurrence-free survival (B) experience of the non-metastatic cohort of 54 patients where follow-up data were available. OS and RFS were estimated with a Kaplan-Meier estimator. The grey-shaded area represents the 95% confidence interval. The risk table below the graph shows numbers of OS and RFS events occurring within the respective interval report in round brackets.

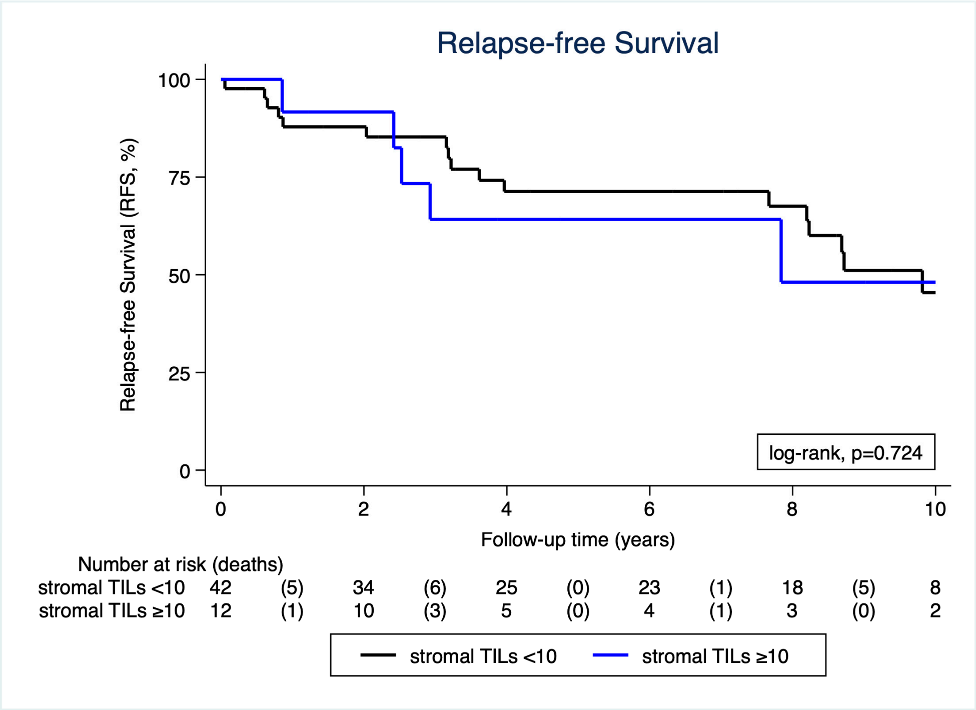

Supplementary Figure S2. Relapse-free survival for sTILs with a cut-off of 10% in the cohort of 54 patients.

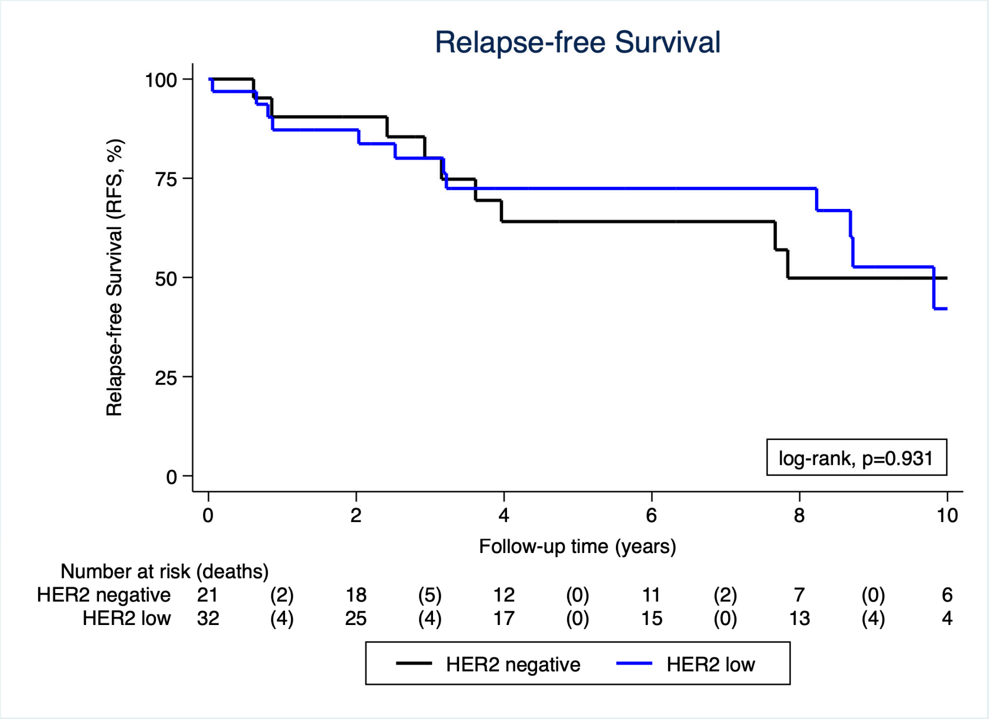

Supplementary Figure S3. Relapse-free survival for HER2-negative and HER2-low tumors in the cohort of 54 patients.
